# Artificial humic substances improve microbial activity for binding CO_2_

**DOI:** 10.1016/j.isci.2021.102647

**Published:** 2021-05-24

**Authors:** Chunyu Tang, Yuelei Li, Jingpeng Song, Markus Antonietti, Fan Yang

**Affiliations:** 1 Joint laboratory of Northeast Agricultural University and Max Planck Institute of Colloids and Interfaces (NEAU-MPICI), Harbin 150030, China; 2 School of Water Conservancy and Civil Engineering, Northeast Agricultural University, Harbin 150030, China; 3 Max Planck Institute of Colloids and Interfaces Department of Colloid Chemistry, 14476 Potsdam, Germany

**Keywords:** chemistry, surface chemistry, Geomicrobiology, microbiology, agricultural science

## Abstract

Humic substances (HS) are an indicator of fertile soils, but more and more soils keep losing there humic matter. This is mostly due to anthropogenic over-cultivation. Artificial humic acid (A-HA) and artificial fulvic acid were synthesized from agricultural litter, with high similarity to natural HS extracted from soil. These samples were added to black soils, and soil activity and nutrients availability were analyzed. The results demonstrate that the content of dissolved organic matter and total organic carbon (TOC) largely increased. The increase in TOC 28 days after addition of A-HA was 21.4 g/kg. This was much higher than the amount of the added A-HA carbon, which was 0.3 g/kg. As a “secondary” benefit, nutrient availability is increased, promoting the growth of plants.

Using high-throughput sequencing we revealed that A-HA strongly supports the growth of photosynthetic *Rubrivivax gelatinosus*, which induced the carbon sequestration. Thus, application of artificial HS shows potential for biologically amplified carbon sequestration within black soils.

## Highlights

• Artificial humic acid and fulvic acid were synthesized from agricultural litter
• With long-term culture, C increment was up to 70 times the chemically added dose
• High-throughput sequencing shows that *Rubrivivax gelatinosus* induces C sequestration
• Application of artificial humic substance induces massive secondary photosynthesis

## Graphical abstract

**Figure:**
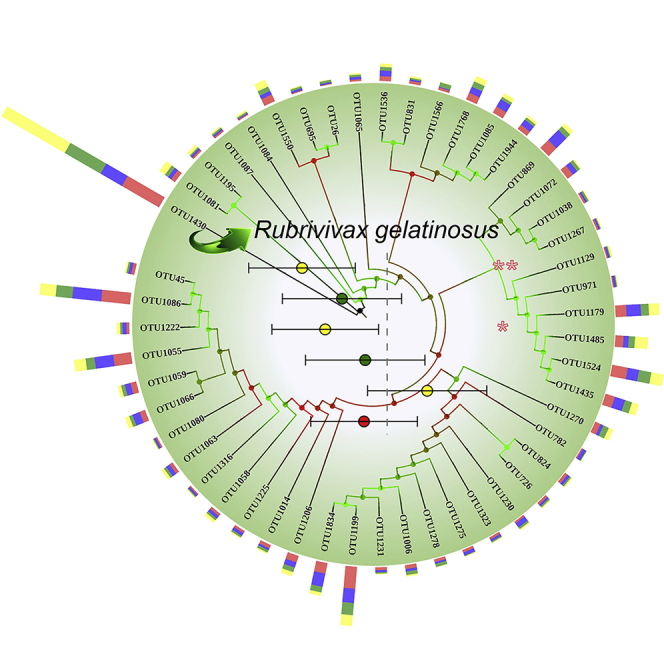

## Introduction

Humic substance (HS) is the product of humification, the second largest process after photosynthesis in the aspect of terrestrial carbon cycle (Hedges and Oades, 1997), and is considered by most to be a resistant substance, which is converted from living organic matter by chemical-microbiological functions (Lehmann and Kleber, 2015). Owing to the polymer character with chemical functionalities such as carboxyl, phenolic, carbonyl, quinone, and methoxy groups (Myneni et al., 1999), HS is recognized by many soil scientists and agronomists as the most important component of a healthy fertile soil (Brady et al., 2016; Nardi et al., 2017). The role of HS in soil improvement has been extensively studied, e.g., altering soil structure (Piccolo et al., 1997), enhancing water holding capacity (Cihlář et al., 2014), maintaining nutrients (Zhu et al., 2018), facilitating microbial activity (Guo et al., 2019), boosting soil carbon pool (Myneni et al., 1999), and more.

On the other hand, and important for the present discussion, HS is also the most promising carbon sink to mitigate atmospheric CO_2_. Hardly known to the general public, the amount of organic carbon in the upper layers of farmed soil is reported as 1,500 Gt (Erickson, 2016), by far more carbon than the atmosphere (currently 870 Gt) or the carbon bound in land plants (550 Gt [Loomis, 1949; Yang et al., 2021b]). Consequently, HS, as a vital component of soil organic matter, is one of the most important players in the global carbon cycle (Shan et al., 2010), and a change of its content on global farming areas has the potential to cure the climate crisis completely. More research efforts have determined the role of HS on sequestrating carbon in soil, whether taking advantages of physical, chemical, or biological actions. For example, Valenzuela et al. (2017, 2019, 2020) and Valenzuela and Cervantes, 2020 systematically studied the performances of HS and related microorganisms on mitigating greenhouse gas emissions, especially CH_4_ and N_2_O in wetlands. A series of investigations revealed that HS not only serves as an electron acceptor or donor for oxidizing CH_4_ or reducing N_2_O but also functions as inert minerals to enhance carbon sequestration. However, few studies have looked at the variation of bacterial communities involved in fixing CO_2_ under the condition of HS, especially those involved in the Calvin cycle.

A technology toward artificial humic substance (A-HS) can thereby have an impact on global climate change (Lal, 2004). Nearly all HS samples used in previous studies to analyze the effects on soil improvement and plant growth were extracted from lignite, commercial HS, or compost (Lulakis and Petsas, 1995; Piccolo et al., 1993; Tahir et al., 2011), i.e., they were natural products. This has obvious restrictions due to regional and seasonal diversity, thereby missing comparability, the lack of effective utilization schemes, and missing sustainability. In previous work, we presented a one-step hydrothermal humification technology (Yang et al., 2019a) and successfully synthesized artificial humic acid (A-HA) and artificial fulvic acid (A-FA) in short times from typical omni-available agricultural wastes as precursors and practically completed carbon yield. Such a sustainable synthetic-chemical route advances the use of waste biomass (e.g., in composting a majority of carbon is metabolized and re-liberated as CO_2_ and CH_4_) and reduces the loss of potential resources. In the first follow-up article, this new A-HA was also already shown to solubilize otherwise insoluble P species (Yang et al., 2019b) and has shown positive effects in simple pot planting experiments (Yang et al., 2019a; Zhang et al., 2020), whereas the specific benefits of typical A-HA and A-FA on the most important biochemical processes in black soils were not analyzed in greater detail. The present study will quantify changes within the carbon pool, including total organic carbon (TOC) and dissolved organic carbon (DOC). At the same time, bacterial abundance and the change of carbon sequestration bacteria associated with the Calvin cycle will be analyzed.

## Results and discussion

Originally, we specially compared the results through principal-component analysis (PCA) to intuitively understand the effects of different HA and FA contents on soil carbon sequestration and nutrient availability (Figure S1). As presented in Figure S1, the proportion of variance accounted for by the first two axes is 75.9%. PCA results visually revealed which index of soil property changes and allowed visualizing the relationship between the increase of TOC and DOC and agriculture effect, total exchangeable base (TEB), and microbial effect in different treatments and cultivation period. Intuitively, TOC, DOC, bacterial abundance, migration distance of available phosphate (AP), and TEB, which have vectors longer than this radius, indicated more contribution to the ordination. According to the results of PCA, we proposed that A-HS have capability to help bind larger amounts of CO_2_ by improving microbial activity in soil and increase the availability of nutrients. Next, we will make a concrete analysis and discussion of the above-mentioned variables in turn.

### Carbon sequestration and variation of TOC and DOC

**Figure 1:**
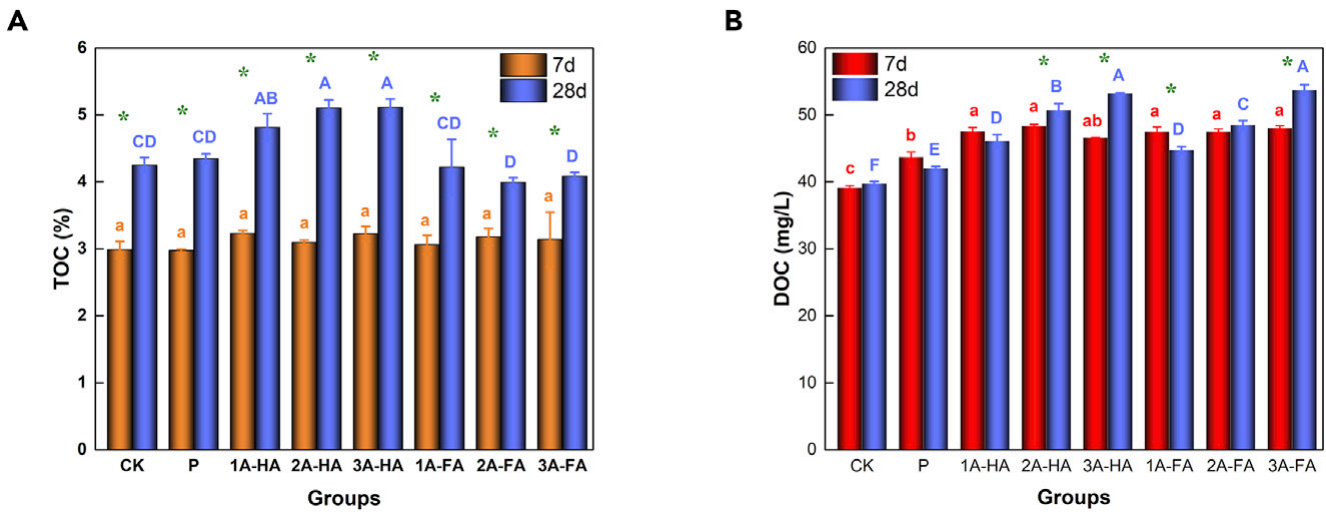
Results of TOC and DOC content in different treatments and cultivation periods (A) TOC content. (B) DOC content. The weight of A-HA and A-FA added was 150 mg/kg (1A-HA/1A-FA), 300 mg/kg (2A-HA/2A-FA), and 450 mg/kg (3A-HA/3A-FA). Statistical repeat bars are for n = 3 repeats, Data are represented as mean ± SEM, “∗” represents that the results show significant differences among different cultivation periods.

TOC and DOC are measured to quantify the diverse carbon contents and to follow the metabolic activity in soils (Howard and Howard, 1990). The contents of TOC and DOC were determined for the different treatments and cultivation periods, and the data are displayed in Figure 1. The values of TOC were measured again, but could also be calculated from the natural carbon content of the soil (2.88 wt %) plus the added A-HA and A-FA. Comparison with the control group (CK) and phosphate fertilizer-treated group (P) reflects that the added carbon is indeed only little; it can be seen that the TOC essentially did not change at shorter cultivation (7-day) (p > 0.05). After 28-day cultivation, TOC in general increased by 1.1–2.2 wt %, more for A-HA than for A-FA (p < 0.05) (Figure 1A). DOC is also the main parameter for evaluating soil fertility as it reflects active components of biological processes (organic nutrients, metabolites, living matter), which is long known (Howard and Howard, 1990). DOC contents significantly increased in all A-HA and A-FA treatments already after 7- and 28-day cultivation (p < 0.05), and the higher the primary A-HA and A-FA concentrations, the higher the DOC. We observed a, nevertheless, impressive improvement of up to 17.0% for 3A-HA and 24.1% for 3A-FA treatment after 28-day cultivation (Figure 1B).

Certainly, TOC (including DOC) in closed compartments can only increase or decrease due to biological activity, that is, photosynthesis (CO_2_ uptake), metabolization (CO_2_ liberation), or microbial apoptosis (Crowther et al., 2019; Liang et al., 2019). The experimentally determined TOC increase is at most 70 times more than the calculated carbon increase in the form of A-HA. The addition of A-HS thereby evokes a giant biological amplification within the black soil biosystem as such (no higher plants involved). As well known, autotrophic bacteria in black soils like nitrifying bacteria, sulfur bacteria, or *Rhodobacteria* are able to fix carbon from simple carbon dioxide gas (CO_2_) (Elbert et al., 2012). Indeed, numerous investigations have already proved that HA can promote the growth and activity of soil autotrophic bacteria (Vallini et al., 1997; Visser, 1985). Our specific observation is that we can amplify this effect, also with respect to the reference experiments, by addition of A-HS. It has been observed that 0.03% of added A-HA carbon gives a biologically amplified reward of up to 2.1 wt % (Table S4) (the specific calculation process is as Equations 1 and 2). This is to be set in context with the “4 per1000” target discussed earlier. The quantification of biomass growth can also be expressed in more common agricultural unities: the soil microbial growth amounts, recalculated from tube to flat geometry, to about 20 tons fixed C ha^−1^ year^−1^ and 28 days, whereas higher plants have great difficulties to grow beyond 10 t C ha^−1^ year^−1^ (Kelliher et al., 2010). As previously reported, other organic amendments also are capable of enhancing the content of soil organic carbon (SOC). For instance, continuous addition of 60 t ha^−1^ year^−1^ pig and cow manure compost increased SOC content (30.0 g kg^−1^) by 3.7g/kg compared with the addition of 30 t ha-1 year^−1^ pig and cow manure compost (26.3 g kg^−1^) under the condition of maize cropping. Compared with no compost addition, the actions of 60 t ha^−1^ year^−1^ and 30 t ha^−1^ year^−1^ pig and cow manure compost increased C to 14.1 g kg^−1^ and 10.4 g kg^−1^, respectively (Wu et al., 2020). Wu et al. (2020) continued to add glucose (labile carbon, a single addition is equivalent to 2% of the initial SOC) to these soils after fertilization treatments to observe soil carbon balance. The results show that SOC content always decreased regardless of whether glucose was added once or repeatedly. Additionally, a field experiment by Zhang et al. (2017) revealed that the application of wheat straw-derived biochar with fertilization strikingly promoted the content of SOC from 8.1 g kg^−1^ to 17.6 g kg^−1^. Obviously, A-HA has a stronger ability to improve SOC (21.4 g kg^−1^), compared with other organic amendments.

(Equation 1) $$\text{Carbon increase in the form of A-HA (\%):}\;S_{1}=A\times C\text{\%}$$

(Equation 2) $$\text{Experimentally determined TOC increase (\%):}\;S_{2}=C_{2}-C_{1}$$

where A and C% represent the proportion of 3A-HA addition (450 mg/kg) and the carbon content in 3A-HA (66.50%), respectively, and C_1_ and C_2_ represent soil TOC content before and after the experiment.

As plants and microbial biomass form a complex interactive ecosystem, microbes contribute nutrients, molecules, and also P and N content to the plant (as, for instance, seen with nitrogen fixing bacteria), whereas plants deliver (also by dead plant mass) metabolites for the growth of bacteria, including carbon sources, nitrogen sources, and phosphorus sources. We therefore rely on perpetuated growth balance, which will set up new equilibria depending on usage, access to light, climate, and higher plants, data which are well beyond the current tube experiments. We rely for carbon fixation on a perpetuated conversion in the soil microbiome system to create a significant fraction of more persistent soil carbon species. A pragmatic statement compressed these findings into “humic acid autocatalyzes formation of more humic acid” (Swift, 2001), which gives hope for the resilience of this mechanisms, i.e., A-HA-reinforced soils are then self-supporting biogeochemical systems.

### 3D-EEM fluorescence spectra and synchronous fluorescence spectra of DOM

**Figure 2:**
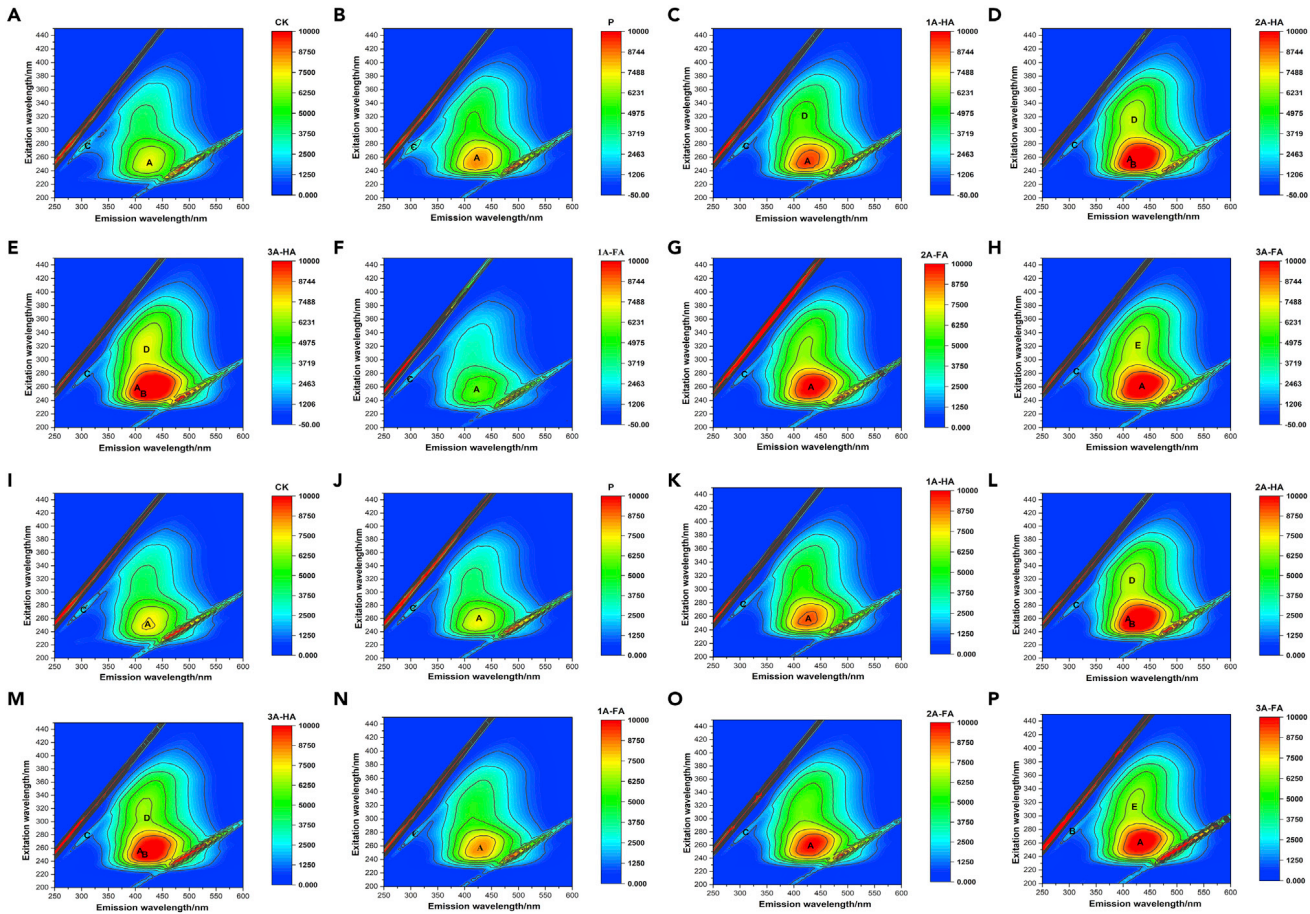
Three-dimensional EEM fluorescence of DOM in different treatments and cultivation period (A–H) CK, P, 1A-HA, 2A-HA, 3A-HA, 1A-FA, 2A-FA, and 3A-FA treatments after 7-day cultivation. (I–P) The same treatments as above, except that the cultivation time has been increased to 28 days

**Figure 3:**
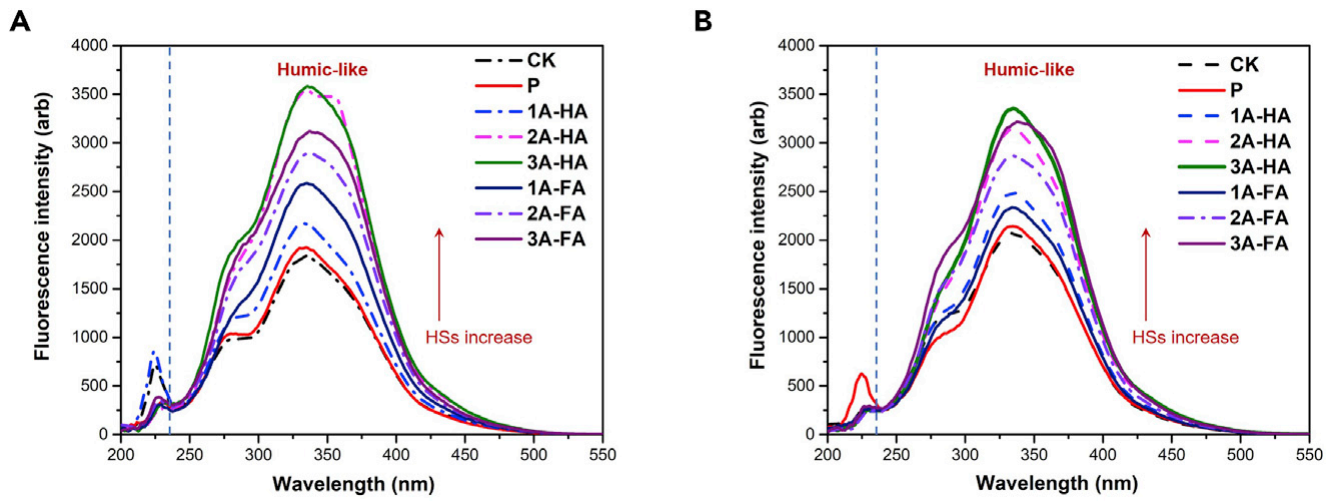
Synchronous fluorescence spectra (SFS) of DOM in different treatments and cultivation period (A) The results of SFS after 7 days. (B) The results of SFS after 28 days.

A simple way to test further metabolization is optical experiments, here three-dimensional excitation-emission matrix (3D-EEM) fluorescence spectroscopy and synchronous fluorescence spectra (SFS) as presented in Figures 2 and 3, respectively. The development of fluorescence is bound to higher aromaticity of a carbon species, thereby higher condensation and defunctionalization and in consequence lower degradation tendency.

Five main peaks, signed as peaks A, B, C, D, and E, can be identified from the DOM fluorescence spectra in the diverse experimental soils (details are displayed in extended data Tables S1 and S2). Peak A is usually assigned to a mixture of HA-like fluorescence (Chen et al., 2003). Peak B is attributed to FA, whereas peak C describes soluble microbial product fluorescence (Chen et al., 2003). Peaks D and E are again all related to HA-like fluorescence (Chen et al., 2003). Conjecturally, there may be the chance in such experiments to trace transformation process of HA-like and FA-like to other compounds in the dynamics of peak A, peak B, and peak E, However, we only see indications for two minor processes: (1) HA-like substances could be oxidatively fragmented to FA-like compounds at higher concentration (2A-HA and 3A-HA) and (2) FA-like substances could be metabolized and later humified into HA-like structures at all FA concentrations.

The quantitative results of SFS on twice diluted DOM extracts are shown in Figure 3. Only one major peak at 330 nm is found, which can be assigned to the HA-like substances. The increase of fluorescence intensity is consistent with the increment of TOC in experimental black soils, i.e., addition of A-HS to soil indeed promotes generation of more HS, here seen in the fraction of extractable HS. In fluorescence, the increase is up to a factor of 2, which must be biologically, as the chemically added (KH_2_PO_4_) dose was 0.1 relative, the most.

### Bacterial diversity and quantitative analysis

**Figure 4:**
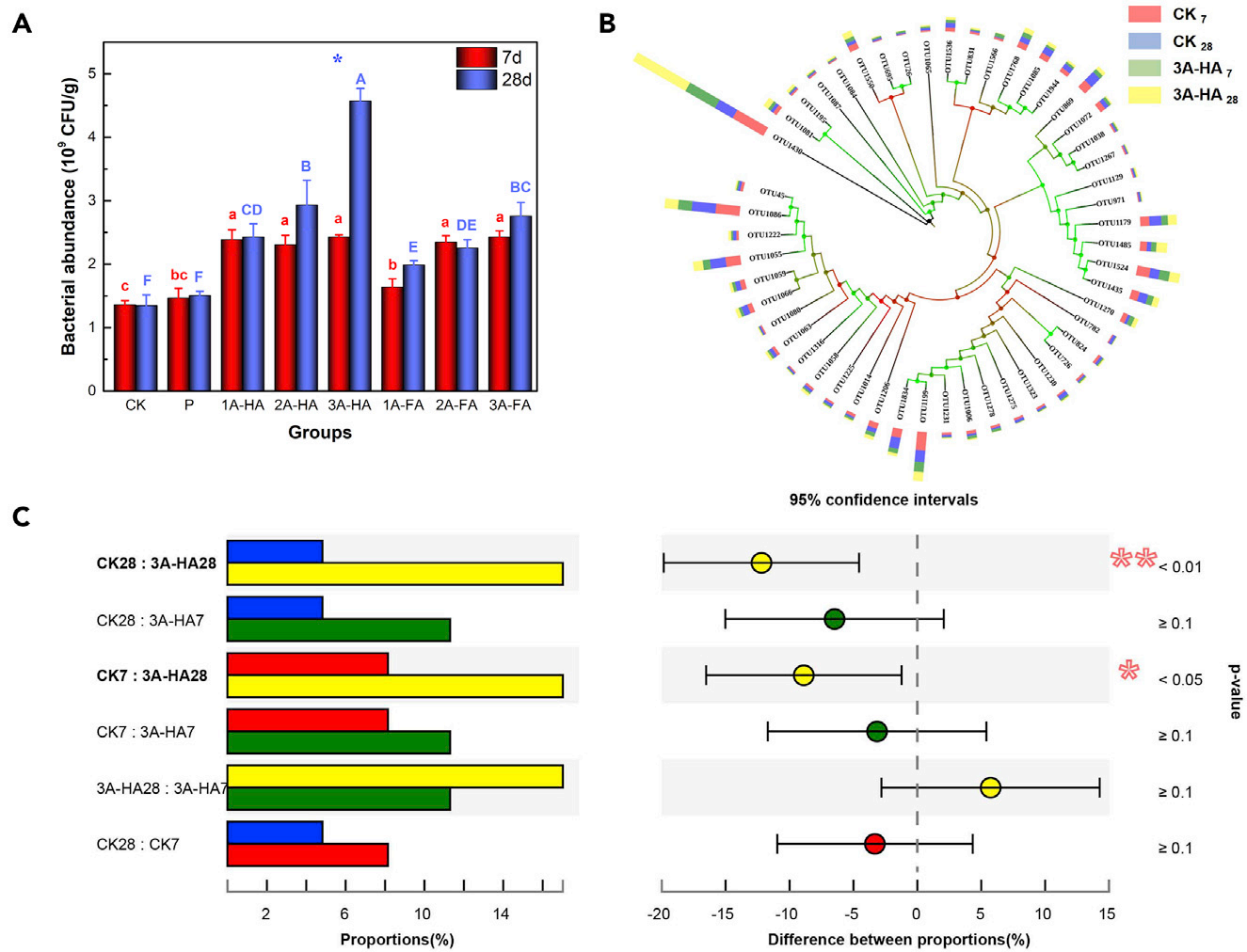
The relevant results of high-throughput sequencing (data are represented as mean ± SEM) (A) The abundance of bacteria; statistical repeat bars are for N = 3 repeats. (B) The community composition and taxonomic information of dominant soil carbon sequestration bacteria with *cbbL* genes. (C) The post-hoc test of OTU1430 with CK_7_, CK_28_, 3A-HA_7_, and 3A-HA_28_ groups, subscript numbers represent the period of cultivation.

We then evaluated changes of bacterial abundance, at first by a flat colony counting method. All A-HS treatments promote the growth of bacteria in black soils, and bacterial abundance after 28-day cultivation is significantly higher than that after 7-day cultivation in 3A-HA treatment (p < 0.05) (Figure 4A). The reason is that A-HS can serve as a source of carbon, nitrogen, and energy for the soil microflora (Lipczynska-Kochany, 2018); enhance the metabolic processes in cells; act as electron transfer catalysts in cellular respiration; and much more (Lovley et al., 1996).

On the other hand, we analyzed particularly changes of communities and biomass of carbon sequestration bacteria involved in the Calvin cycle (Berg, 2011) to verify if growth of carbon sequestration bacteria explains the increase of soil carbon content. Soil samples of CK and 3A-HA group (the dataset with the most pronounced differences) were separately analyzed. High-throughput sequencing was applied to analyze the diversity of the bacteria. At 97% sequence similarity level, operational taxonomic units (OTU) numbers in the experimental groups of CK and three times artificial humic acid (3A-HA) in different cultivation varied from 711 to 843, and the taxonomic information of dominant carbon sequestration bacteria is listed in Figure 4B. Also, the relative abundance of OTU1430 increased from 11.3% (7 days) to 17.0% (28 days) in 3A-HA treatment, whereas CK had 8.3% and 4.9%, respectively. We also conducted the post-hoc test (Tukey-Kramer) of OTU1430 in each group (CK_7_, CK_28_, 3A-HA_7_, and 3A-HA_28_) (Figure 4C). The detailed results demonstrate that OTU1430 massively changes in the community structure before and after the experiment, especially between CK_28_ and 3A-HA_28_ (p < 0.01).

Aside from high-throughput sequencing, quantitative real-time PCR (qPCR) was utilized to quantify the abundance of bacterial *cbbL* genes (the gene encoding Rubisco and CO_2_ fixing) in four soil samples, but no significant change in the abundance of *cbbL* genes of four soil samples (p > 0.05) (Table 1) was found.

**Table 1:** The abundance of bacterial *cbbL* genes in four soil samples

	CK_7_	CK_28_	3A-HA_7_	3A-HA_28_
*cbbL* quantity (10^9^ copies/g)±	1.43 ± 0.37^a^±	1.42 ± 0.33^a^±	0.98 ± 0.32^a^±	1.07 ± 0.36^a^

The same letter indicates that there was no significant difference between the experimental groups.

a A-HA: artificial humic acid.

A comparison of the genes according to the Basic Local Alignment Search Tool (Blast) shows that OTU1430 has a high relative similarity with *Rubrivivax gelatinosus*, a carbon sequestration bacterium (the homology is up to 89.58%; the results of multiple sequence alignment are presented in Figure S2).

The results obtained from the analysis of bacterial diversity and quantitative revealed that the relative abundance of OTU1430, i.e., *R. gelatinosus* (a facultative phototrophic nonsulfur bacterium), changed greatly during the whole experiment, with DOC reflecting the actual activity, whereas TOC integrating over all activity and the following humification of dead species. It was reported that *R. gelatinosus* can grow in a variety of environments, including in the dark using a variety of inorganic carbon/organic acids to conduct chemoautotrophic/heterotrophic growth pattern, as well as in photoheterotrophic growth patterns depending on organic matter as carbon source and hydrogen donor (Maness et al., 2002; Ouchane et al., 1997). As a result, multiple carbon sequestration pathways are available, and we even could speculate how A-HA and A-FA support the growth of *R. gelatinosus*.

### Enhancement of nutrient availability

**Figure 5:**
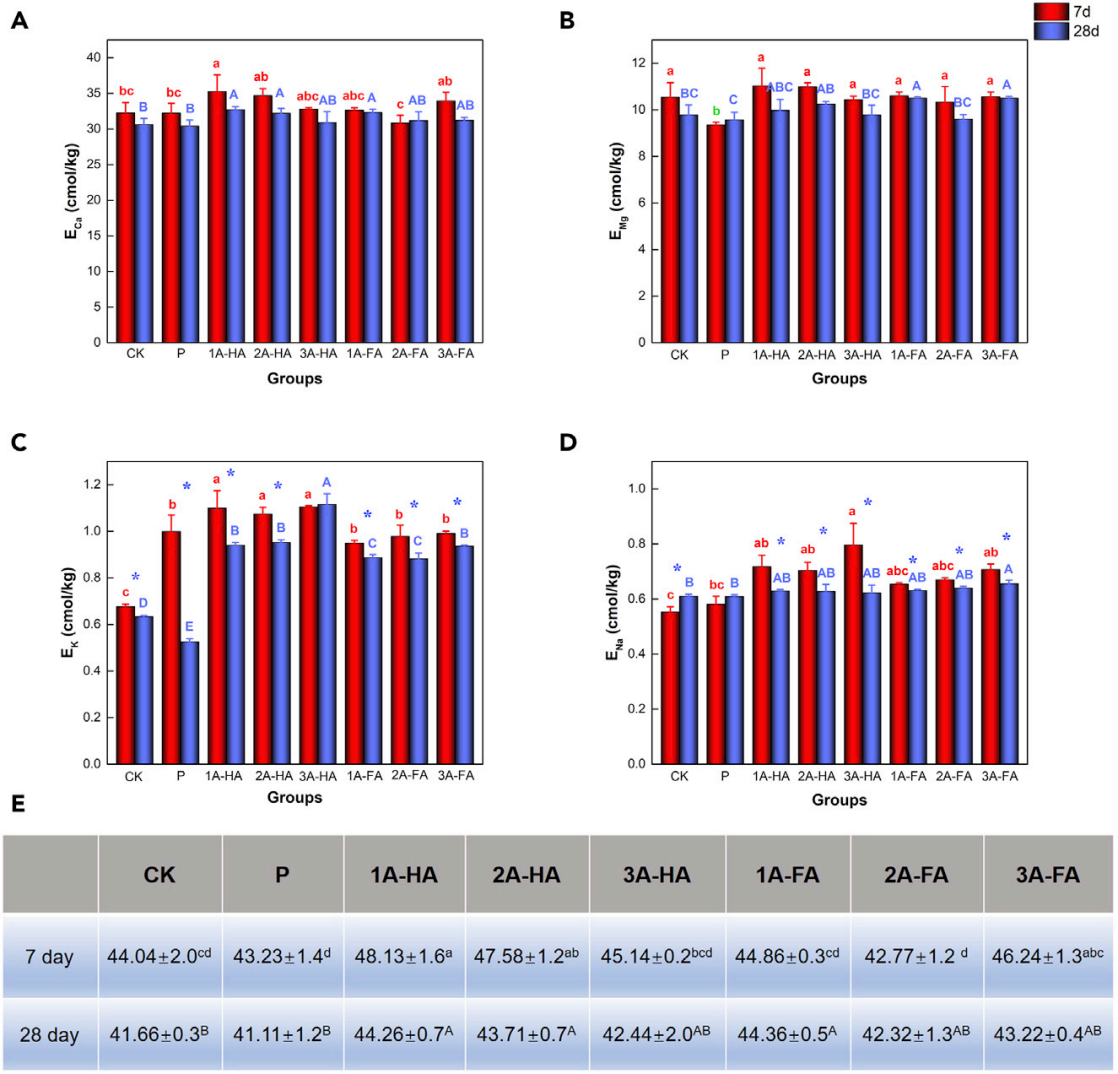
Results of exchangeable case base content in different treatments and cultivation period; statistical repeat bars are for n = 3 fully repeated experiments (data are represented as mean ± SEM, “∗” represents that the results show significant differences among different cultivation periods) (A) The content of Ca_e_. (B) The content of Mg_e_. (C) The content of K_e_. (D) The content of Na_e_. (E) The content of TEB.

**Figure 6:**
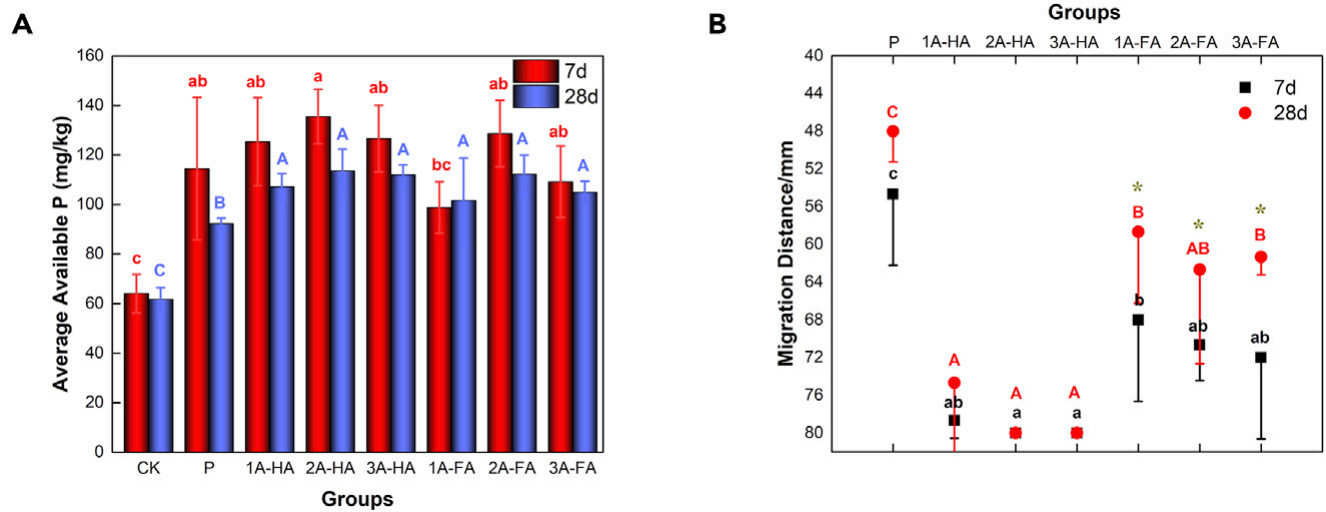
Results of AP concentration and vertical migration distance in different treatments and cultivation period (A) AP concentration. (B) Vertical migration distance of AP. The same letter represents that there was no significant difference between different treatments in the level of 0.05; on the contrary, different letters represents that there was a significant difference between different treatments in the level of 0.05. In addition, ∗ represents that there was a significant difference between two cultivation periods with the same treatment. Statistical repeat bars are for n = 3, each.

The enhancement of soil nutrient content by A-HS may also be one of the vital causes for biological amplification of carbon content. Consequently, we also measured exchangeable base cations and AP. The detailed results are all listed in (Figures 5 and 6).

Figure 5 presents the changes in the four exchangeable base cations (Ca_e_, Mg_e_, K_e_, Na_e_) and TEB. Ca_e_ and Mg_e_ were all positively correlated with TEB, not only in 7-day cultivation (r = 0.976, p < 0.001; r = 0.761, p < 0.05) but also in 28-day cultivation (r = 0.965, p < 0.001; r = 0.842, p < 0.01) (Figure S3).

Exchangeable bases not only maintain soil nutrients and buffer soil acidification but also provide nutrients needed for plant growth (Yang et al., 2021b). Values of Ca_e_, Mg_e_, K_e_, and Na_e_ and TEB content are displayed in Figure 5 to grasp the effect of A-HA and FA on TEB. The content of Ca_e_ after A-HA treatment (Figure 5A) was significantly higher than that of all other treatments after 7-day cultivation (p < 0.05), and the content of Ca_e_ in 1A-HA, 2A-HA, and 1A-FA treatments after 28-day cultivation were apparently higher than that in other treatments (p < 0.05). However, there was no significant change between two different cultivation periods in the same treatment (p > 0.05). We take this as a reflection of the ability of HA and FA as organic acids to etch and solubilize minerals (Yang et al., 2019a). The change (Figure 5B) of Mg_e_ content is less significant, both after 7- and 28-day cultivation. The content of K_e_ in A-HA-treated soils (Figure 5C) significantly improved. The content of Na_e_ (Figure 5D) improved after A-HS addition but changed the most in 3A-HA treatment after 7-day cultivation (p < 0.05). TEB content (Figure 5E), the sum of Ca_e_, Mg_e_, K_e_, and Na_e_, improved with all its components.

TEB and in general cation exchange capacity are meaningful to evaluate soil fertility (Sinore et al., 2018). Cations are kept in an easily exchangeable form (Brady et al., 2016) because of the carboxyl and phenolic hydroxyl groups of HS (Perminova et al., 2005). Previous investigations on the structure of A-HS revealed that it contains the same chelating and ion-binding groups, hence the addition of A-HS quantitatively increases the content of exchangeable cations in black soils, further improving soil fertility (Klein et al., 2018; Plaza et al., 2006; Tipping, 2002). We can confirm this effect at addition levels of only 0.01%–0.03% added carbon. Therein, the promotion role of A-HA is better than that of A-FA on TEB already after short times.

As shown in Figure 6A, there is only little difference in AP in the presence or absence of A-HS after 7-day cultivation (p > 0.05). However, the concentration of AP in A-HS-treated black soils significantly increases when compared with the blinds after 28-day cultivation (p < 0.05). A comparison of the AP migration distance is shown in Figure 6B. AP vertical migration distance in the series of A-FA_7d_ treatments increases from 54.7 mm to 68.0 mm, 70.7 mm, and 72.0 mm with increasing added A-FA addition, respectively (p < 0.05). Interestingly, the AP vertical migration distance in the series of A-HA_7d_ treatments increased even more, in spite of having higher molecular weight. After 28-day cultivation, A-HA preserves the vertical distribution of AP, whereas AP “migration” decreased significantly for A-FA when compared with the 7-day experiments (p < 0.05), which means that the available AP is consumed and not repleted.

P is one of the most important elements in the biosphere and agriculture, especially for promoting plant growth (Quanxian et al., 2008). However, excessive use of phosphate fertilizers induces remineralization and P accumulation in soils; only a small and variable fraction of the added P is directly available to the plants (Van der Salm et al., 2017). It is because soil contains a large amount of iron and aluminum oxides, and once applied phosphate fertilizer enters the soil, it will be fixed to form insoluble P (Yang et al., 2021a). The application of A-HS is only able to slow down the conversion of phosphate fertilizer to insoluble P or form ternary system with Fe-Al oxides and phosphorus complex to maintain its solubility. Unavoidably, partly inorganic P was fixed by Fe-Al oxides. In the present study, the application of A-HA and A-FA indeed promoted increasing AP concentration and vertical migration distance, and the addition of A-HA provides best performance. Consequently, we could show that application of already 0.03 wt % of A-HS improved the accessibility of AP in the soil itself and in a sustainable manner as it did not deplete along the giant microbial biomass growth.

### Conclusion

The so-called 4 per 1000 initiative delineated at the Paris climate agreement (2016) that an increase by 0.4 wt % of soil carbon in farmed areas every year is enough for a complete cure of the CO_2_ crisis (Soussana et al., 2019). The calculation is simple: taking China only with its 1.4 million square kilometers of arable land and adding to the 10-cm top-layer 4 permille carbon, China would bind about 600 Mt C while counteracting soil degradation. We will see below that with biological amplification, this value can even reach 3 Gt/a.

To conclude, we can state that A-HS, made from biomass litter in a rapid and efficient engineering process, can be used to strengthen the carbon pool, the microbiome, and thereby the nutrient availability even in already strong soils. In the context of carbon sequestration in farmland, it is a central observation that we found the TOC increased not only by the added dose (here between 0.1 and 0.3 permille) but also due to biological amplification effects to up 2.1 wt %, already after 28 days of model cultivation. This carbon fixation power is much higher than the one of higher plants and could, with high statistical significance, be assigned to *R. gelatinosus* and its dependent microbial cluster community.

Such treatments not only directly increase the SOC content of black soils but also indirectly strengthen the fertility of soils through microorganic and photochemical reactions. Beside the increase of carbon content, we could also follow increased availability of nutrients, which for A-HA was even persistent. The effect of A-HA on modified soils was superior to that of A-FA, especially for high concentrations. We assume that this relates to the higher pH and redox buffer capacity of the corresponding humic acids, which stabilize a healthy soil microbiome, in our examined range along the added dose.

Applying intelligent humic acid synthesis technology and using such biological nonlinearities, the next generation of farmed soils might indeed do what the Paris climate protocol predicted: to become a major player in fighting the climate change.

### Limitations of the study

The work mainly focused on the investigation of the increased SOC and the variation of soil carbon sequestration bacteria involved in Calvin cycle, and amino sugars, specific indices for fungal and bacterial residues, were not measured, to verify the contribution of microbial residues to the accumulation of stable SOC.

## STAR★methods

### Key resources table

**Table:** 

REAGENT or RESOURCE	SOURCE	IDENTIFIER
**Deposited data**		

High-throughput sequencing data from four soil samples in triplicate	This paper; Mendeley Data	https://doi.org/10.5061/dryad.18931zcv4

**Oligonucleotides**		

cbbl gene: primer K2fF (5’-ACCAYCAAGCCSAAGCTSGG-3’); V2fR (5’-GCCTTCSAGCTTGCCSACCRC-3’)	This paper	N/A

**Software and algorithms**		

R language	Borcard et al., 2018	https://www.r-project.org/
SPSS	This paper	N/A
Origin	This paper	N/A

### Resource availability

#### Lead contact

Further information and requests for resources should be directed to and will be fulfilled by the lead contact, Fan Yang and Markus Antonietti (yangfan_neau@163.com; Markus.Antonietti@mpikg.mpg.de).”

#### Materials availability

This study did not generate new unique reagents.

#### Data and code availability

Original source data for Figures 4B and 4C in the paper is available (https://doi.org/10.5061/dryad.18931zcv4).

### Method details

#### Nomenclature of modified soils

Pure soil experiments-omitting plants were conducted. The experimental soil is hereafter labeled as only P, 1A-HA ~ P, 2A-HA ~ P, 3A-HA ~ P, 1A-FA ~ P, 2A-FA ~ P, 3A-FA ~ P, respectively, where the prefix number of A-HA/FA represents the amounts of additions. Additionally, neither P nor A-HA, A-FA were added in the blank incubation, labeled as CK. All treatments were cultured for 7 and 28 days, respectively, and the experiments were replicated three times. The properties of experimental soil and HS samples are shown in Tables S3 and S4.

#### Preparation of experimental soil and A-HS

Black soil sample used in this study was obtained from topsoil 0–15 cm of a cultivated layer in a field located Horticultural Culture Station in Northeast Agriculture University, Harbin, China (45°44 ′22 ″N, 126°43 ′30 ″E). The soil was air-dried and ground to pass a 2-mm sieve. Carbon (C), hydrogen (H), and oxygen (O) content in soils were measured by elemental analyzer (Flashsmart). Particle size composition was examined by using a Mastersizer 3000 laser diffractometer and classified by American Soil Society classification. The main physical and chemical properties of black soil were listed in Table S3.

Additionally, A-HA and A-FA applied in the present study were all synthesized by HTH (Yang et al., 2019a) along previously reported hydrothermal recipes. The content of C, H, O, N of A-FA was also measured by elemental analyzer (Flashsmart). Meanwhile, the content of total acidity and typical functional groups were measured by Ba(OH)_2_ method (Brooks and Sternhell, 1957) and Ca(CH_3_COO)_2_ method (Schnitzer and Gupta, 1965), respectively. The properties of A-HA detected by Yang et al. (Yang et al., 2019a) and instant-detected A-FA were summarized in Table S4.

#### Batch soil column experiments

The containers used in the present study which were made of acrylic plexiglass replaced wax columns used by Khasawneh and Soileau (Khasawneh and Soileau, 1969). The height and diameter of plexiglass column are 100 mm and 50 mm, separately. Each plexiglass column was closed at the bottom with two pieces of gaze to prevent soil loss. 200 g dried soil was homogenized and packed into each column, in order to achieve a bulk density of 1.27 g/cm^3^. To standardize the moisture in the soil, the container was placed in a water-filled box, so that the water entered the soil from bottom to top. The bottom of the packed columns was covered with parafilm to prevent moisture loss, and the tops of the packed columns were also covered with parafilm to prevent moisture evaporation but not to prevent air flow. To the end, the packed columns were allowed to equilibrate for 48 h at 25°C prior to experiment.

After equilibration, experimental groups were treated by the joint conduct of reagent grade phosphate fertilizer (KH_2_PO_4_) dissolved with deionized water, and A-HA or A-FA dissolved with 2 mL 3% NH_4_OH were homogeneously sprinkled on top of the samples. In sum, the amount of KH_2_PO_4_ was 0.136 g/column, which is equivalent to 5.000 μmol P/g soil. The amounts of A-HA and A-FA added were 150 mg/kg, 300 mg/kg and 450 mg/kg soil in each column, respectively, corresponding to approximately 0.100, 0.200, 0.300 mg/g C in A-HA and 0.092, 0.183, 0.275 mg/g C in A-FA, separately. The columns were then incubated vertically under the conditions of 25°C, and the light incubator was always ventilated. In order to observe the variation of bacteria involved in Calvin cycle under A-HS treatments, including photosynthetic bacteria and aerobic chemoautotrophic bacteria, we set 12 hr light and 60% solar light intensity. In addition, the added A-HS was sterilized to minimize the impact of foreign microorganisms on the experimental soil microbial community. To exclude the disturbance by NH_4_OH which is applied to dissolve HS and thus treat the soil homogeneously, 3% NH_4_OH ~ P was also applied as a reference treatment. It was found that the addition of 2 mL 3% NH_4_OH had only slight effects on soil properties. As a result, these data are only displayed in Table S5.

#### Culture and counting of bacteria

The surface planting technique, modified from Estermann and McLaren (Estermann and McLaren, 1961), was taken up to cultivate bacterial of experimental black soils. Bacteria culture medium, which was applied to cultivation bacteria abundance in the present study, i.e., beef extract peptone medium purchased from Solarbio, is consistent with that applied by Gu, et al. (Gu et al., 2007) and Yao, et al. (Yao et al., 2008). Briefly, 1 g of each soil sample was removed and dissolved in 10 mL culture medium, then gently shaken at 125 rpm for 30 min. The supernatant was diluted to 10^−5^, 10^−6^ and 10^−7^ in the medium and coated on the plate, eventually. Bacteria were cultured at 37°C for 24 hr and then counted. Flat colony counting method (Gao et al., 2011) was used to determine bacterial abundance in experimental black soils. The number of CFU at each dilution rate was counted after incubation and the average CFU/g in experimental soils was determined.

#### Analysis of cbbL genes

Amplification of cbbL gene along with genomic DNA for DNA extraction, PCR amplification, high-throughput sequencing and quantitative real-time PCR (qPCR) were done by using primer K2fF (5′-ACCAYCAAGCCSAAGCTSGG-3′) and V2fR (5′-GCCTTCSAGCTTGCCSACCRC-3′). All samples were sent to the Majorbio Bio-pharm Technology Co., Ltd (Shanghai, China) for high throughput sequencing and qPCR, and each sample was detected in triplicate. The data were analyzed on the free online platform of the Majorbio Cloud Platform (www.Majorbio.com)

#### Characterization

For the determination of TOC, DOC, DOM, exchangeable base cations and bacterial abundance, three mixed soils in each column were taken for measurement and averaged as the results. TOC was determined by measuring the organic carbon content in a Vario TOC tube (Plante et al., 2011). For the determination of DOC and the fraction of DOM, 1 g soils were extracted by 0.01 mol/L CaCl_2_ and shaken at 25°C and 145 rpm for 24 hr. As followed, the extract of mixture was filtered by the membrane (0.45 μm) via filtration. The content of DOC was determined by a Vario TOC tube, as same as the determination of TOC. The DOM was identified by fluorescence spectrophotometer (F-7100FL). EEM spectra could be collected in the scan ranging from 250 to 600 nm at increments (1 nm) for emission and from 200 to 450 nm at 5 nm increments for excitation. The wavelength of excitation from 200 to 550 nm with a constant offset was used to gain SFS (Δλ = Em- Ex = 60 nm). All the fluorescence measurements scanning speed were set at 1200 nm min^−1^.

Additionally, each sample was sectioned from the top into 20 slices with every slice 4 mm thickness using a sharp knife after removing the gauze and parafilm. These sliced samples were used to measure the vertical migration the average content of AP in each soil column. AP presenting in soils was determined by Olsen method and extracted with 0.5 mol/L NaHCO_3_ solution (Olsen, 1954) and then determined with ascorbic acid-ammonium molybdophosphate blue method (Murphy and Riley, 1962) in an UV spectrophotometer (UV-1800 TC). The exchangeable cations of Ca, Mg, K, and Na were extracted by 0.1 mol/L BaCl_2_ compulsive exchange method (Gillman and Sumpter, 1986) and then measured by atomic absorption spectrophotometer (ICE-3500) (Hendershot and Duquette, 1986), and the sum of Ca_e_, Mg_e_, K_e_ and Na_e_ content was recognized as TEB content (Borůvka et al., 2009).

Tests of homogeneity of variance were conducted, and separate one-way (ANOVAs) were performed to test for differences in seven treatments. Least significant difference (LSD) was used to make post-hoc comparisons between different treatments. Differences were considered significant at p < 0.05. Statistical analyses were conducted using SPSS 19.0 software. The plots of EEM fluorescence spectra and SFS were all drawn by Origin Pro (2016). PCA which was examined relationships among several quantitative descriptors or variables in the present study was performed by R 3.4.4 software (Borcard et al., 2018).
