# Supplementary material for: Artificial humic substances improve microbial activity for binding CO2
Source: iScience. 2021 May 24;24(6):102647. doi: 10.1016/j.isci.2021.102647 (PMC8387571; doi:10.1016/j.isci.2021.102647)
Supplement: Document S1. Figures S1–S3 and Tables S1–S5 [file mmc1.pdf]

iScience, Volume *24*

Supplemental information

Artificial humic substances improve microbial activity for binding CO_2

Chunyu Tang, Yuelei Li, Jingpeng Song, Markus Antonietti, and Fan Yang

Principal component analysis (PCA)

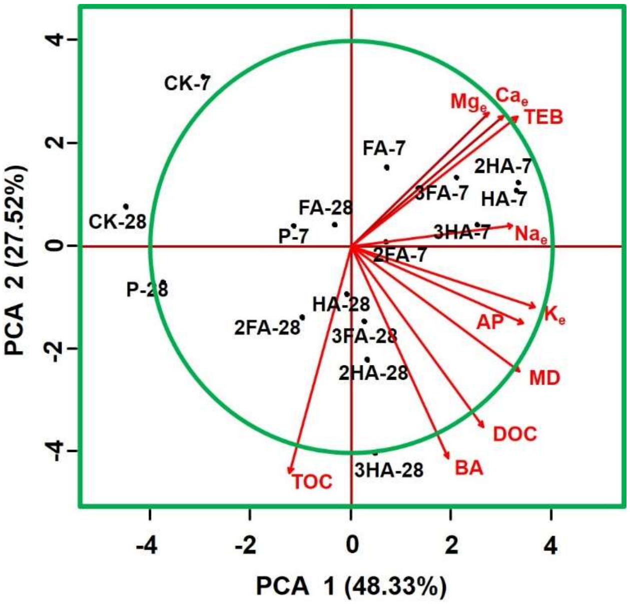

Figure. S1 Principal component analysis of variables in different A-HS treatments and cultivation period, Related to Figure 1, 5 and 6 (MD: migration distance; BA: bacteria abundance; AP: available phosphorus)

Multiple sequence alignment

```
OTU1430    CCTGTCCGGTCGCAACTACGGTCGCGTGATCTACGAGGGC     40
Sbjct      CCTGTCGGGCCGCAACTACGGCCGCGTGATCTACGAAGGC     40
Consensus  cctgtc gg cgcaactacgg cgcgtgatctacga ggc

OTU1430    CTCAAGGGCGGCCTCGACTTCATGAAGGACGACGAGAACA     80
Sbjct      CTGAAGGGCGGGCTGGACTTCATGAAGGACGACGAGAACA     80
Consensus  ct aagggcgg ct gacttcatgaaggacgacgagaaca

OTU1430    TCAACTCGCAGCCGTTCATGCACTGGCGCGACCGCTTCCT    120
Sbjct      TCAACTCGCAGCCCTTCATGCACTGGCGTGACCGTTTCCT    120
Consensus  tcaactcgcagcc ttcatgcactggcg gaccg ttcct

OTU1430    CTACGTCATGGACGCCGTCAACAAGGCGAGCGCGGCGACC    160
Sbjct      CTACGTGATGGACGGCGTGAACAAGGCCAGCGCCGCCACC    160
Consensus  ctacgt atggacg cgt aacaaggc agcgc gc acc

OTU1430    GGCGAGGTGAAGGGCTCGTACCTGAACATCACGGCGGCGA    200
Sbjct      GGCGAGGTCAAGGGCAGCTACCTGAACATCACCGCCGCGA    200
Consensus  ggcgaggt aagggc   tacctgaacatcac gc gcga

OTU1430    CGATGGAGGACATGTACGAGCGCGCGGAGTTCGCCAAGCA    240
Sbjct      CGATGGAGGACATGTACGAACGTGCCGAGTTTGCGAAGGA    240
Consensus  cgatggaggacatgtacga cg gc gagtt gc aag a

OTU1430    ACTCGGCTCGATCGTCGTGATGGTCGACCTCGTAGTCGGC    280
Sbjct      ACTGGGTTCGATCGTCGTCATGGTCGACCTGGTGATCGGC    280
Consensus  act gg tcgatcgtcgt atggtcgacct gt  tcggc

OTU1430    TGGACCGCGATCCAGTCGATCGCGAACTGGGCGCGCAAGC    320
Sbjct      TGGACCGCGATCCAGAGCATCGCCAACTGGGCGCGCAAGA    320
Consensus  tggaccgcgatccag   atcgc aactgggcgcgcaag

OTU1430    ACGACATGATCGTGCACATGCACCGCGCCGGCCACGGCAC    360
Sbjct      ACGACATGCTGGTGCACATGCACCGCGCCGGCCACGGCAC    360
Consensus  acgacatg t gtgcacatgcaccgcgccggccacggcac

OTU1430    CTACACGCGGCAGAAGAACCACGGCGTCAGCTTCCGCGTG    400
Sbjct      CTACACGCGGCAGAAGAACCACGGCGTCAGCTTCCGCGTC    400
Consensus  ctacacgcggcagaagaaccacggcgtcagcttccgcgt

OTU1430    ATCGCGAAGTGGCTGCGCCTCGCGGGCTGCGACCACCTCC    440
Sbjct      ATCGCCAAGTGGCTGCGCCTGGCCGGCTGCGACCACCTGC    440
Consensus  atcgc aagtggctgcgcct gc ggctgcgaccacct c

OTU1430    ACACCGGCAC                                  450
Sbjct      ACACCGGCAC                                  450
Consensus  acaccggcac
```

Fig. S2. The results of multiple sequence alignment between OTU1430 and *R. gelatinosus*, Related to Figure 4 (The homology is up to 89.58%)

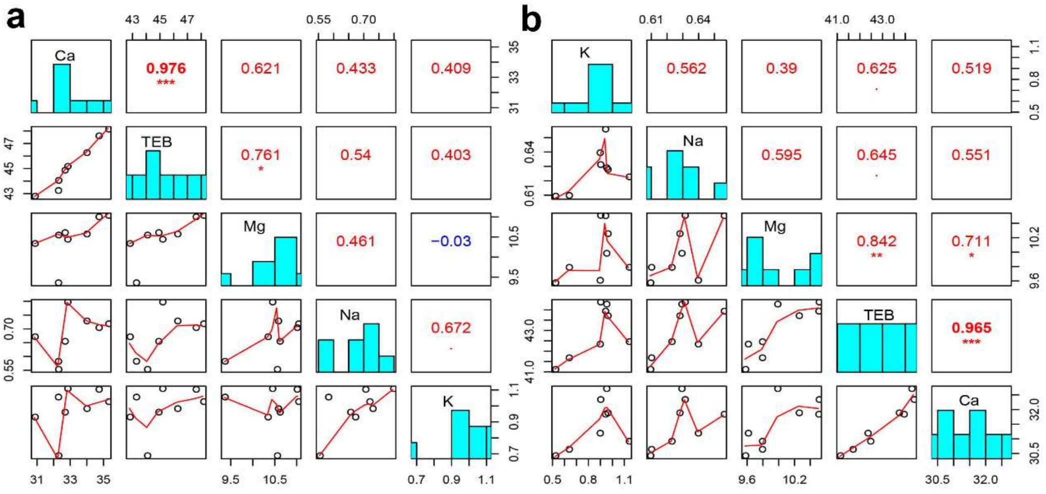

Fig. S3. Person correlation matrix of Ca_e, Mg_e, K_e, Na_e and TEB, Related to Figure 5 (a: 7-day cultivation period; b: 28-day cultivation period. In addition, * represents a significant correlation at the 0.05 level; ** represents a significant correlation at the 0.01 level; *** represents a significant correlation at the 0.001 level)

Tables

Table S1 The fluorescence wavelength and intensity of different fluorescent substances extracted from experimental black soils with different treatments in 7-day cultivation, Related to Figure 2

7-day	CK	P	1A-HA	2A-HA	3A-HA	1A-FA	2A-FA	3A-FA
Peak	255/425	250/420	250/425	250/405	250/400	255/425	255/420	250/415
A	7351	8567	9451	> 9999	>9999	8545	>9999	>9999
Peak	/	/	/	245/410	245/410	/	/	/
B	/	/	/	>9999	>9999	/	/	/
Peak	280/310	280/310	280/310	280/310	280/310	280/310	280/310	280/310
C	3264	3737	2568	2383	2370	2356	2333	2262
Peak	/	/	310/415	310/415	310/415	/	/	/
D	/	/	5695	6902	7296	/	/	/
Peak	/	/	/	/	/	/	/	320/420
E	/	/	/	/	/	/	/	6900

Table S2 The fluorescence wavelength and intensity of different fluorescent substances extracted from experimental black soils with different treatments in 28-day cultivation, Related to Figure 2

28-day	CK	P	1A-HA	2A-HA	3A-HA	1A-FA	2A-FA	3A-FA
Peak A	250/425<br>7701	255/425<br>7358	255/425<br>9164	250/405<br>> 9999	250/410<br>>9999	255/425<br>8545	260/430<br>>9999	255/420<br>>9999
Peak B	/<br>/	/<br>/	/<br>/	245/410<br>>9999	245/415<br>>9999	/<br>/	/<br>/	/<br>/
Peak C	280/310<br>2739	280/310<br>2730	280/310<br>2531	280/310<br>2415	280/310<br>2492	280/310<br>2603	280/310<br>2392	280/310<br>2350
Peak D	/<br>/	/<br>/	/<br>/	310/415<br>6859	310/415<br>6514	/<br>/	/<br>/	/<br>/
Peak E	/<br>/	/<br>/	/<br>/	/<br>/	/<br>/	/<br>/	/<br>/	320/420<br>6528

Table S3 Properties of soil samples as experimental, Related to STAR METHOD

C (%)	H (%)	O (%)	Particle size composition		
			Clay (< 2 nm)	Silt (2~50 nm)	Fine sand (>50 nm)
2.88	0.67	5.31	18.32%	60.41%	21.27%

Table S4 Composition and acidity of A-HA and A-FA samples, Related to STAR METHOD

A-HS	C (%)	H (%)	O (%)	N (%)	Total acid (mmol/g)	-COOH (mmol/g)
A-HA	66.50	6.05	21.64	1.63	7.33	2.83
A-FA	61.02	5.08	32.93	0	3.50	1.62

Table S5 Values of different variables of experiment black soil in NH_4OH ~P treatment (MD: migration distance; BA: bacterial abundance), Related to STAR METHOD

Variables	TOC (%)	DOC (mg/L)	AP (mg/L)	MD (mm)	Ca_e (cmol/kg)	Mg_e (cmol/kg)	K_e (cmol/kg)	Na_e (cmol/kg)	BA (10^9 CFU/g)
7d	3.2±0.1	45.2±0.2	115.7±5.3	78.6±1.9	35.3±2.3	11.0±0.8	1.0±0.0	0.8±0.1	1.5±0.2
28d	4.5±0.1	45.1±0.4	103.6±3.7	50.7±7.5	32.7±0.4	10.0±0.5	0.9±0.0	0.6±0.0	1.7±0.3
